# Efficacy of adjuvant radiotherapy for treatment of adrenocortical carcinoma: a retrospective study and an updated meta-analysis

**DOI:** 10.1186/s13014-020-01533-3

**Published:** 2020-05-24

**Authors:** Jiawei Zhu, Ziye Zheng, Jing Shen, Xin Lian, Zheng Miao, Jie Shen, Fuquan Zhang

**Affiliations:** Department of Radiation Oncology, Peking Union Medical College Hospital, Chinese Academe of Medical Sciences & Peking Union Medical College, Beijing, 100730 China

**Keywords:** Adrenocortical carcinoma, Adjuvant radiotherapy, Surgery, Meta-analysis

## Abstract

**Background:**

Adrenocortical carcinoma (ACC) is a rare and highly aggressive malignancy. Surgical resection is the standard treatment for localized ACC, but the local recurrence remains high. Adjuvant radiation (ART) has been proposed as a means to reduce recurrence rates in ACC after surgery with conflicting results from nonrandomized studies. We performed a retrospective study and a meta-analysis to determine the impact of ART on survival outcomes.

**Methods:**

A retrospective study of the adrenocortical cancer database in Peking Union Medical College was conducted. We selected postoperative ACC patients with or without ART. A meta-analysis is also performed to compare the outcomes between ART and only surgical resection in ACC patients. The obtained data were analyzed using SPSS 23.0 and Stata 15.0 statistical software. Differences between two groups were compared using the log-rank test for retrospective analysis and estimated by calculating the odds ratio (OR) and 95% confidence interval (CI) for meta-analysis.

**Results:**

Of a total of 75 patients available in the database, 12 patients underwent postoperative ART and were matched one to one to patients with only surgical resection. There was no significant difference on overall survival between ART group and control group (log-rank *P* = 0.149). Locoregional recurrence was diagnosed in 2 of the ART group, and in 4 of the control group (*P* = 0.64). A total of 238 participants were selected for the meta-analysis, of which 111 and 127 patients underwent ART after surgical resection and only surgical resection, respectively. Overall survival is significantly higher in ART group, with an odds ratio (OR) of 2.41 (95% CI of 1.33, 4.38; *P* = 0.004). Besides, meta-analysis significantly favored ART for locoregional recurrence-free survival and disease-free survival, with an OR of 4.08 and 2.27, respectively.

**Conclusions:**

Our results show that compared to only surgical resection, ART is an effective postoperative treatment for ACC.

## Background

Adrenocortical carcinoma (ACC) is a rare and highly aggressive malignancy with an incidence rate of 1–2 case per million population [1]. Surgical resection is the standard treatment for localized ACC to achieve a long-term survival [2]. However, locoregional failures are common even after complete surgical resection with estimated rates of local recurrence as high as 30% [3], which was with associated poor long-term survivorship [4]. Distant failures are also quite often, with 42% patients having liver metastases and 39% patients having liver metastases at initial stage or during follow-up [5].

Adjuvant treatments including mitotane and radiotherapy (RT) can extend tumor progression time. Adjuvant radiation (ART) has been proposed as a means to reduce high recurrence rates in ACC following surgery with curative intent [6]. A recent population-based study used the National Cancer Database and found that ART on improved overall survival (OS) with a 40% decreased risk of death in patients with positive margins [6]. Due to the rarity of this disease, there is a paucity of high-quality prospective evidence in academic literature evaluating the role of ART in ACC. Therefore, physicians must rely on the critical appraisal of non-randomized studies with variable patient populations, clinical stage, and outcomes.

In order to improve clinical decision making, we systematically reviewed the literature for studies evaluating the role of ART after primary surgery of ACC, and performed a meta-analysis of oncological outcomes including OS, locoregional recurrence-free survival (LRFS), and recurrence-free survival (RFS). Although previous reviews have investigated this question, our systematic review provides an updated analysis. We excluded single arm studies from our review to provides a current and rigorous review of this question. We also report our clinical experience with ACC by comparing patients who were treated using surgery followed by ART with those using surgery alone.

## Methods

### Institutional paired match comparison

We performed a retrospective search of our adrenocortical cancer database. We selected data from adult patients who were treated using surgery, between 1 January 1994 and 1 January 2014.

The patients were separated into two Groups for analysis based on treatment strategy. The treatment group consisted of individuals treated with surgery followed by ART; the control group consisted of individuals treated with surgery only. The inclusion criteria comprised reported surgical margins, ART performed at our institution. The periods of use and doses given were inconsistent among patients because of variation in tolerance and toxicity. The exclusion criteria comprised patients that underwent ART for the treatment of recurrence or of secondary lesions, intra-operative rupture of the tumor capsule. The required minimal follow-up periods were 1 years after surgery, except for the patients that died before. The two groups were paired for comparison based on age, sex, surgical margin status, tumor site, ki67 index, mitotane, tumor size, and clinical stage according to European Network for the Study of Adrenal Tumors classification guidelines. Conformal three-dimensional radiotherapy was applied at the tumor bed for all patients in the ART group within 2 months after surgery, except one patient had ART within 6 months after surgery. Radiation extension and dose were chosen according to adrenocortical cancer severity (based on margin status, size, stage, and immunohistochemistry).

The groups were compared using OS, LRFS, and RFS outcome variables. Local and distant recurrences were diagnosed using computed tomography magnetic resonance or positron-emitting tomography imaging. The times to local and distant recurrence were considered the periods from the surgery date to the date of the imaging examination that revealed the recurrence. If recurrence did not occur, patients were censored at the date of death or at the date of the last follow-up examination. Two and one patients with oligometastatic disease were included in ART group and control group, respectively. These three patients were excluded from the disease-free survival analysis. OS time was measured from the surgery date to the date of death. Patients still living at the last date of follow-up were censored in the analysis.

The statistical analysis was performed using SPSS 23.0. The two groups were compared using the paired t test for continuous variables and Fisher’s exact test for categorical variables. The Kaplan-Meier method was used for the survival analysis (GraphPad Prism 8.02 for Windows application). The differences between two groups in endpoints were compared using the log-rank test. All tests were 2-sided. A *p*-value < 0.05 was considered to indicate a statistically significant result.

### Meta-analysis for oncological outcomes

This study was conducted and reported according to the Preferred Reporting Items for Systematic Reviews and Meta-Analysis Statement (PRISMA) issued in 2009 [7]. The PRIMA checklist is attached (S1 File). A systematic electronic search of PubMed, EmBase, and the Cochrane Library databases was performed for eligible studies from inception to September 2019. Studies that compared the outcomes between ART and only surgical resection for the treatment of ACC were selected for meta-analysis in this study. There is no language or publication status limitation for literature review. The following index words were used: adrenocortical, carcinoma, cancer, radiation, radiotherapy, irradiation and adjuvant.

The retrieved records were independently reviewed by two investigators. If the investigators disagreed about the eligibility of an article, it was resolved by debating with a third reviewer. The inclusion criteria were as follows: (1) patients with adrenocortical carcinoma; (2) intervention was ART compared with only surgical resection; (3) outcomes data reported; and (4) article type as original research. In the current study, the outcome included tumor outcomes, such as OS, LRFS and RFS.

The Newcastle-Ottawa Scale (NOS) was used to evaluate the quality of the included studies for cohort studies. The NOS was based on the following three subscales: selection (four items), comparability (one item), and outcome (three items). NOS ranges were 0 to 8, and scores of 0–3, 4–6, and 7–8 were considered as low, moderate, and high quality, respectively, for these studies.

Event numbers of oncologic outcomes from both ART and control group were pooled from eligible studies. Meta-analysis was performed using Stata software (version 15.0; Stata Corporation, College Station, TX, USA). Heterogeneity between studies was assessed via a chi-square analysis and the I [8] test, with significance set at *P* < 0.05. Included studies are considered to have low heterogeneity (or be homogeneous) if I [8] is less than 25%, moderate heterogeneity if I [8] is 25 to 50%, and high heterogeneity if I [8] is greater than 50%. If homogeneity existed between studies, meta-analysis was performed with a fixed effect model. If significant heterogeneity was confirmed, either by significant chi-square test (*P* < 0.05) or I [8] greater than 50%, meta-analysis was performed using a random effects model. Lastly, a pooled odds ratio (OR) was performed with 95% confidence interval (CI), and the overall effect was assessed via the z statistic with statistical significance set at *P* < 0.05.

## Results

### Institutional paired match comparison

A total of 75 patients were available to assess from the institutional database. Of these, 12 patients underwent postoperative adjuvant RT at the Peking Union Medical College Hospital. These were matched one to one to patients who did not undergo adjuvant RT suing the propensity matching. Patients underwent resection between 2003 and 2018. All patients had localized or oligometastatic disease and underwent surgery with curative intent. Median follow-up times for the ART and control groups were 23 months (range: 10–76) and 37 months (14–92). respectively.

Table 1 summarizes the baseline characteristics of patients by treatment group. There was no significant difference between the groups in term of sex, age, stage, receipt of mitotane, tumor size, endocrine syndrome, or surgical margin status. The majority of patients treated with adjuvant RT were treated after 2010 (91.7%). There was an average of 51 days from the date of surgery to initiation of RT (range, 16 to 199 days). Only one patient had a more than 100 days interval between surgery and initiation of RT. All patients were treated with three-dimensional conformal RT.

**Table 1:** Baseline Characteristics of Patients

	ART (*n* = 12)	Control (*n* = 12)	*P* value
Sex, n (%)			
Male	5 (41.7)	5 (41.7)	1^a,c^
Female	7 (58.3)	7 (58.3)	
Mean age, y (range)	43.4 (19–70)	42.9 (22–62)	0.924^b,c^
Disease stage			
I	2 (16.7)	1 (8.3)	1^a,c^
II	5 (41.7)	6 (50.0)	
III	3 (25.0)	4 (33.3)	
IV	2 (16.7)	1 (8.3)	
Mitotane use			
Yes	4 (33.3)	3 (25.0)	1^a,c^
No	8 (66.7)	9 (75.0)	
Ki67 index			
< 20%	4 (33.3)	5 (41.7)	0.772^a,c^
≥ 20%	5 (41.7)	3 (25.0)	
Not reported	3 (25.0)	4 (33.3)	
Tumor side			
Right	8 (66.7)	7 (58.3)	1^a,c^
Left	4 (33.3)	5 (41.7)	
Mean tumor size, cm (range)	7.9 (3–15.3)	8.4 (4.5–16.5)	0.688^b,c^
Surgical margins			
Negative	6 (50.0)	8 (66.7)	0.848^a,c^
Positive	4 (33.3)	2 (16.7)	
Not reported	2 (16.7)	2 (16.7)	

^a^Fisher’s exact test

^b^Paired t test

^c^Accounted for in the model calculating the propensity weights for adjuvant RT.

**Fig. 1:**
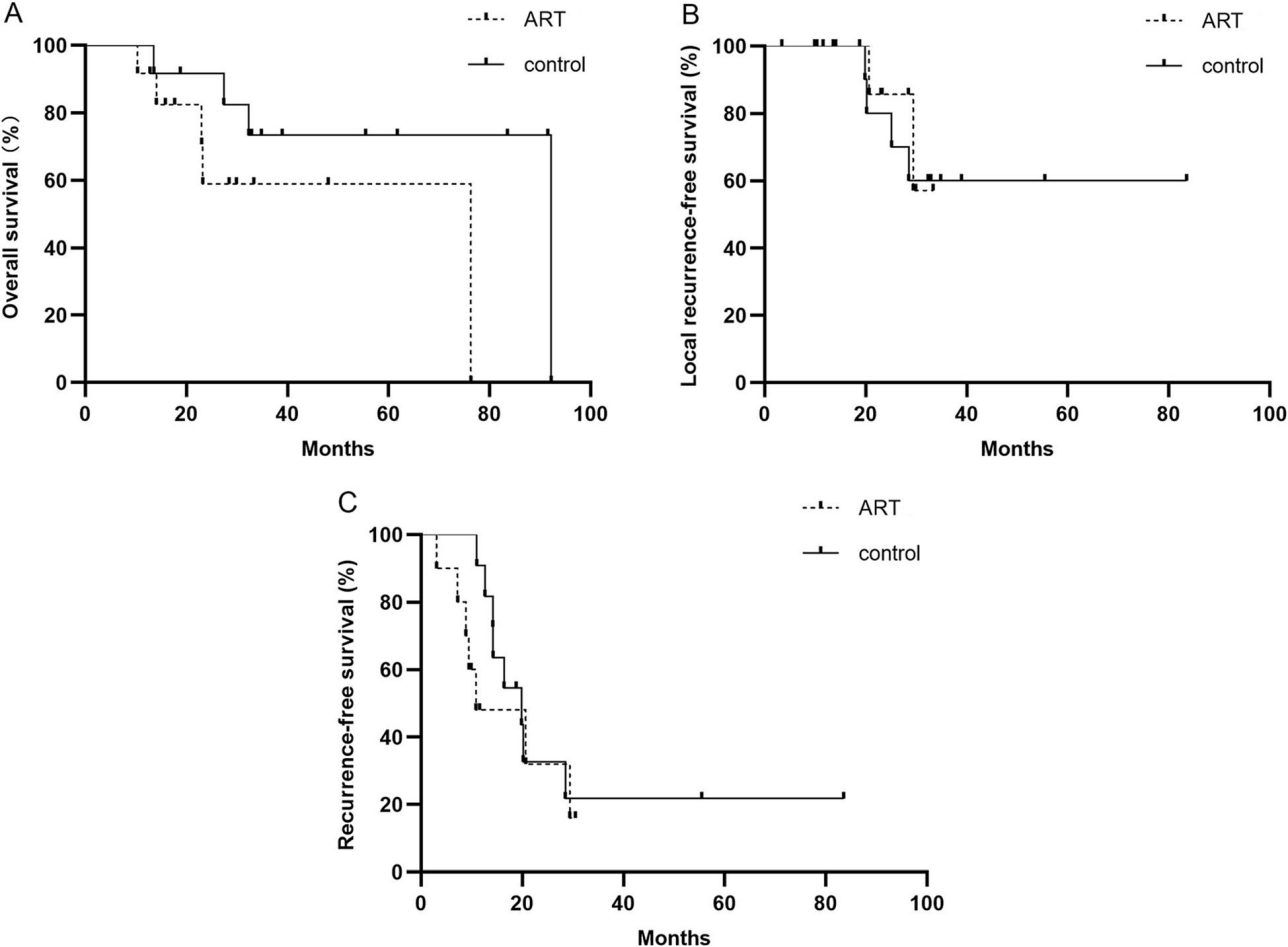
Kaplan-Meier analysis of overall survival (**a**), local recurrence-free survival (**b**), recurrence-free survival (**c**)

The OS distributions for case subjects and control subjects were significantly different (log-rank *P* = 0.149) (Fig. 1a). A total of 9 (58.3%) patients [5 (41.7%) treated with adjuvant RT and 4 (33.3%) treated without adjuvant RT] are known to have died. OS estimates at 3 were 62.7% vs 71.0%, respectively, with an adjusted HR of 2.81 (95% CI, 0.65 to 12.09; *P* = 0.165).

Local recurrence was diagnosed in 2 of the ART group, and in 4 of the control group (*P* = 0.64). Locoregional recurrence-free survival (LRFS) was similar between the two groups (log-rank *P* = 0.879) (Fig. 1b). Locoregional RFS estimates at 3 years were 73.6% vs 56.6%, respectively, with an adjusted HR of 0.87 (95% CI, 0.16 to 4.91; *P* = 0.88).

Any recurrence, including distant failures or death, occurred in 15 (71.4%) patients [7 (70%) treated with RT and 8 (72.7%) treated with resection alone]. There was an insignificant difference in DFS between patients who received adjuvant RT and those who underwent resection only (log-rank *P* value 0.085) (Fig. 1c). DFS estimates at 3 years were 37.5% vs 60.3%, respectively, with an adjusted HR of 2.58 (95% CI, 0.846 to 7.84; *P* = 0.096).

### Meta-analysis for oncological outcomes

**Fig. 2:**
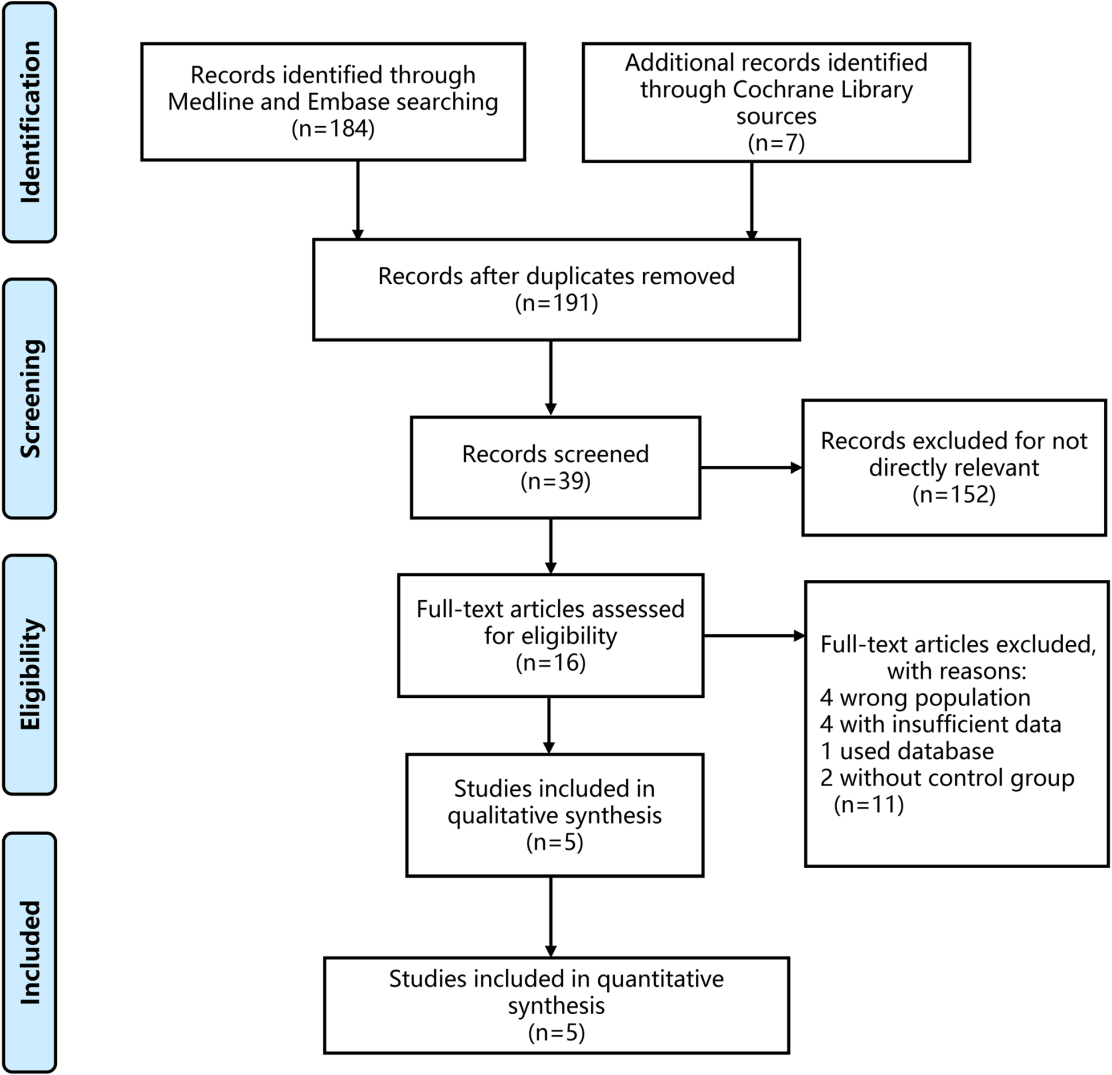
Study selection process of meta-analysis

A total of 191 studies were retrieved based on our searching strategy. After reading the abstract, 16 studies were related to our aims (Fig. 2), and 11 of them were subsequently excluded with reasons given. The remaining 5 studies and our data were selected for our analysis (Table 2) [9–14]. A total of 238 participants were selected for the meta-analysis, of which 111 and 127 patients underwent ART after surgical resection and only surgical resection, respectively. All the studies included were retrospective design and the majority of them were from Europe and America.

**Table 2:** Characteristics of the included studies

Study, year	Country	Study types	Age (RT vs non-RT, years)	Gender (LS vs RT, male %)	RT participants	Non-RT participants	Disease stage	Median follow-up (RT vs non-RT, month)
Fassnacht, 2006 [9]	Germany	Retrospective	43 vs 48	N/A	14	14	I, II, III and IV	37
Habra, 2013 [10]	America	Retrospective	48 vs 44	37.5 vs 34.4	16	32	II and III	22.1 vs 32.2
Sabolch, 2015 [11]	America	Retrospective	49.5 vs 42.3	50 vs 25	20	20	II and III	34
Srougi, 2017 [12]	Brazil	Retrospective	40 vs 38	40 vs 10	10	10	N/A	32 vs 35
Gharzai, 2019 [13]	America	Retrospective	44.9 vs 47.1	46.2 vs 46.2	39	39	I, II, III and IV	50.5
Zhu, 2020	China	Retrospective	43.4 vs 42.9	41.7 vs 47.1	12	12	I, II, III and IV	23 vs 37

The Newcastle-Ottawa Scale (NOS) was used to examine the quality of all included studies. Most studies (5 of 7) were marked as 7 in NOS as they failed to report enough follow-up time. The 1 remaining study received full scores in NOS, indicating they were high quality original studies (Table 3).

**Table 3:** Methodological quality of the studies included in the meta-analysis

First author	Representativeness of the exposed cohort	Selection of the unexposed cohort	Ascertainment of exposure	Outcome of interest not present at start of study	Control for important factors or additional factors	Outcome assessment	Follow-up long enough for outcomes to occur	Adequacy of follow-up of cohorts	Total quality score
Fassnacht	☆	☆	☆	☆	☆	☆	☆	-	7
Habra	☆	☆	☆	☆	☆	☆	☆	-	7
Sabolch	☆	☆	☆	☆	☆	☆	☆	-	7
Srougi	☆	☆	☆	☆	☆	☆	☆	-	7
Gharzai	☆	☆	☆	☆	☆	☆	☆	☆	8
Zhu	☆	☆	☆	☆	☆	☆	☆	-	7

### Overall survival

**Fig. 3:**
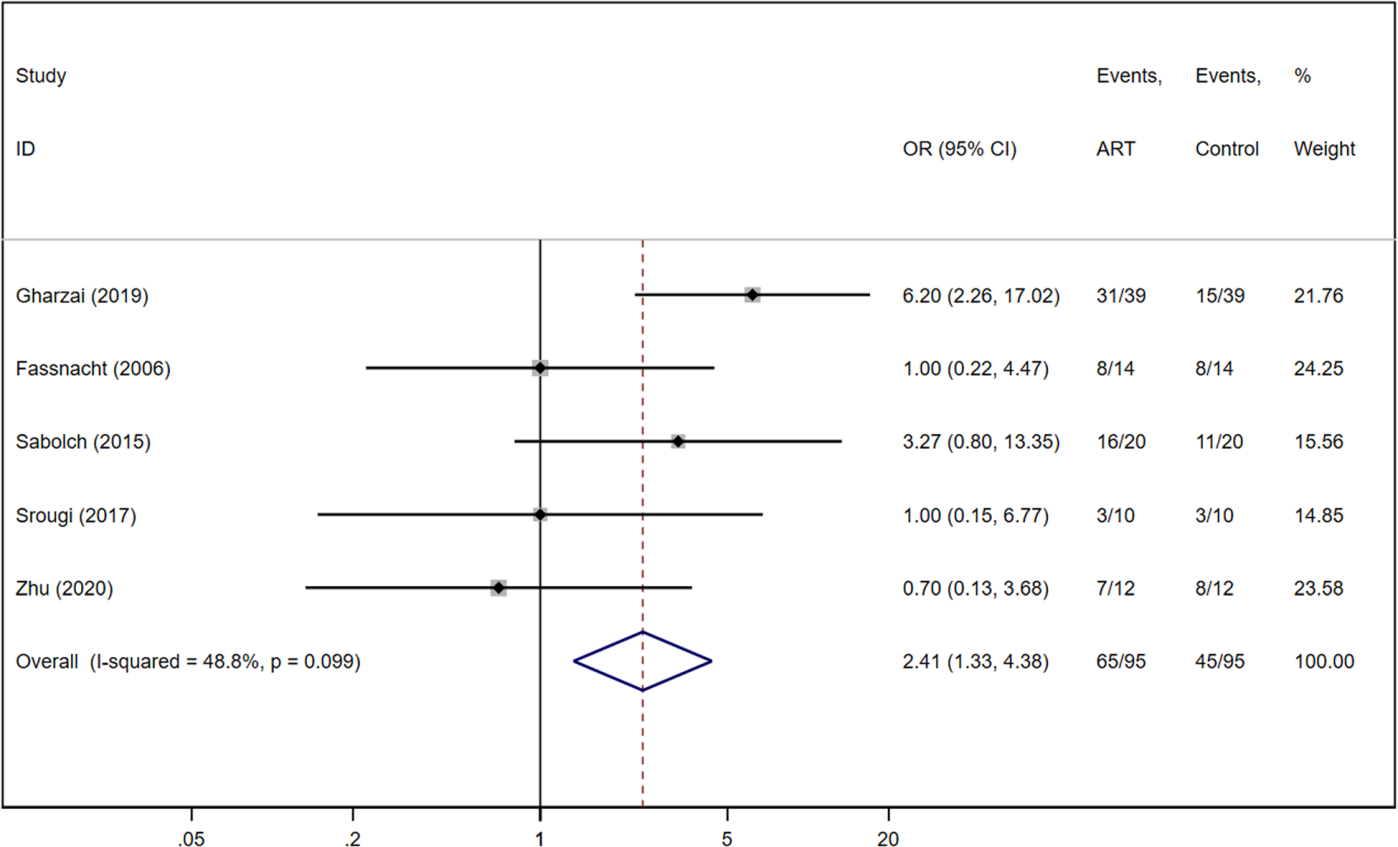
Forest plot of OS for ACC

Of the six studies included in our meta-analysis, five reported OS event numbers for both ART group and control group (Fig. 3). The total patient population was 95 in both groups. There was significant heterogeneity among the five retrospective studies (Chi^2^ = 7.81, *P* = 0.099, I^2^ = 48.8%), and the fixed effects model was applied. Meta-analysis significantly favored OS with ART, with an OR of 2.41 (95% CI of 1.33, 4.38; *P* = 0.004).

### Local recurrence-free survival

**Fig. 4:**
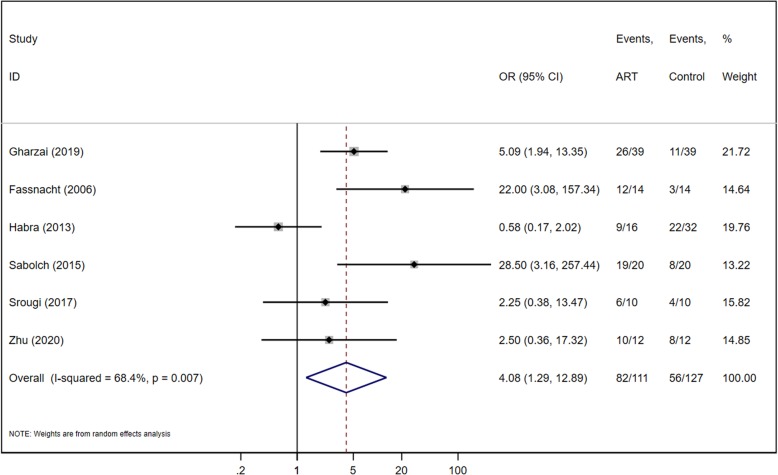
Forest plot of LRFS for ACC

**Fig. 5:**
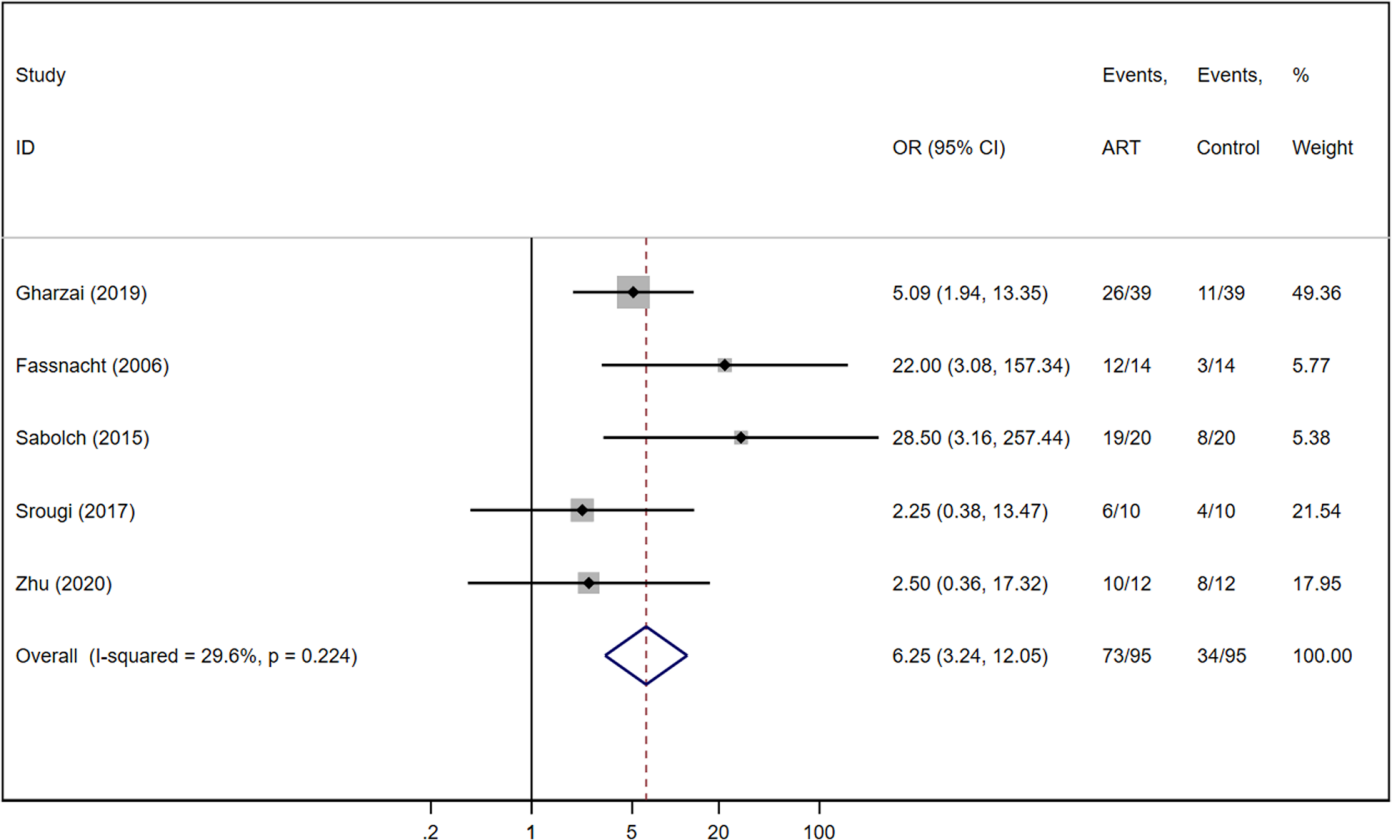
Forest plot of LRFS for ACC after sensitive analysis

All the six studies reported event numbers for LRFS for both ART and control groups (Fig. 4). There were 111 patients in the ART group and 127 in the control group. Meta-analysis revealed significant heterogeneity among the six retrospective studies (Chi^2^ = 15.83, *P* = 0.007, I^2^ = 68.4%), and the random effects model was applied. The pooled analysis significantly favored ART for LRFS of ACC patients, with an OR of 4.08 (95% CI of 1.29, 2.14; *P* = 0.016). After performing a sensitive analysis, and found the heterogeneity was introduced by the MDACC study [10]. The imbalanced sample size and different follow-up time caused the referral bias. After that, combining the results of the five cohorts (190 patients) a statistical significance in favor of ART group was observed with a OR = 6.25 (95% IC 3.24–12.05, *P* < 0.001) (Fig. 5). There was low heterogeneity among the five studies (Chi^2^ = 5.69, *P* = 0.224, I^2^ = 29.6%) indicating that the pooled analysis is valid.

### Recurrence-free survival

**Fig. 6:**
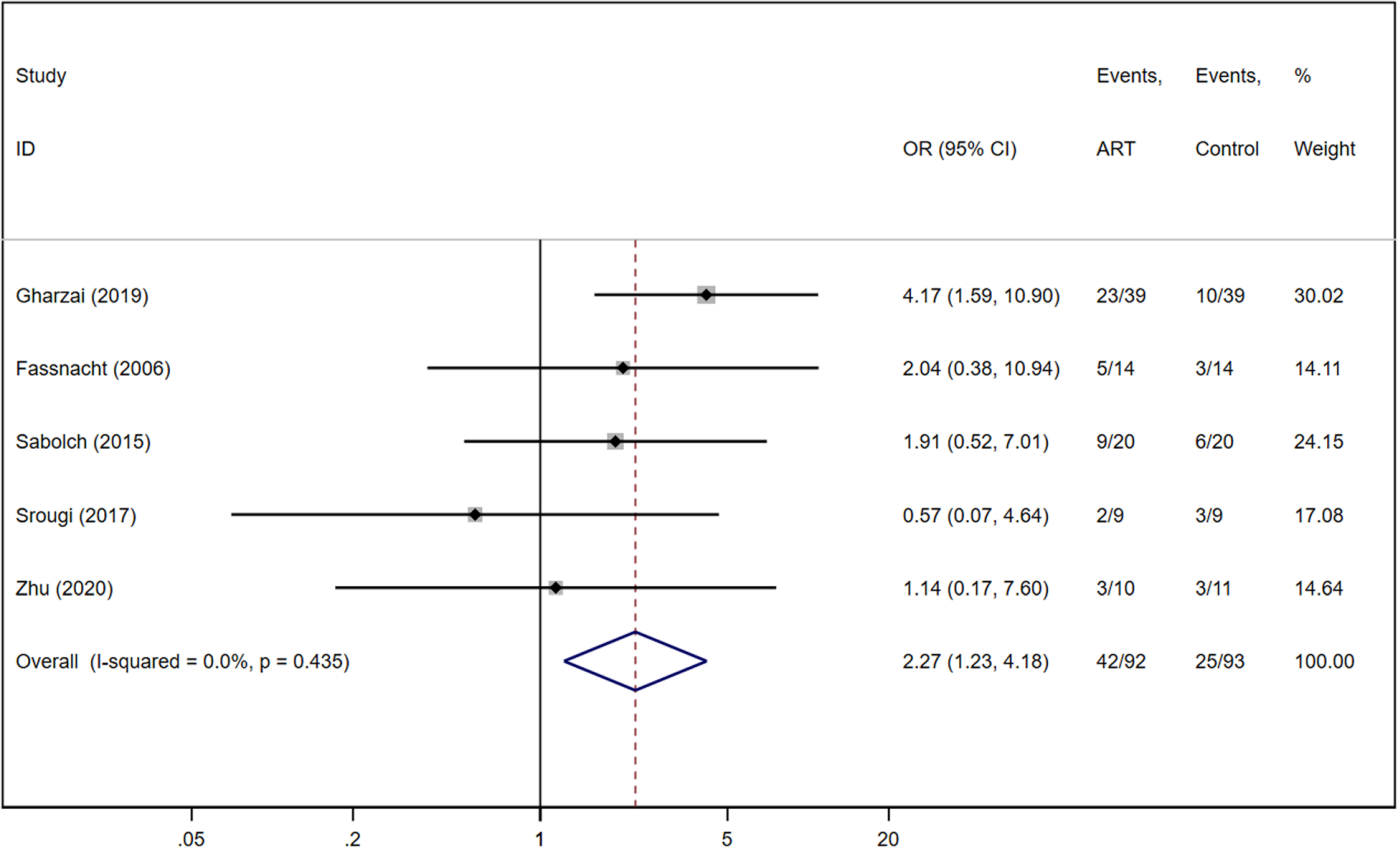
Forest plot of RFS for ACC

Five of six studies reported event numbers for recurrence-free survival for both ART and control groups (Fig. 6). There were 92 patients in the ART group and 93 in the control group. There was low heterogeneity among the five retrospective studies (Chi^2^ = 3.79, *P* = 0.435, I^2^ = 0%), and the fixed effect model was applied. Meta-analysis significantly favored ART for disease-free survival in ACC, with an OR of 2.27 (95% CI of 1.23, 4.18; *P* = 0.009).

## Discussion

ACC was historically thought to be radioresistant due to the mixed results from previous case reports or case series publish between 1958 and 2006 [14–16]. With the application of modern radiation techniques and the publication of high-quality retrospective studies, the view of the role of ART in the treatment of ACC has changed. Besides, the use of mitotane showed to have a beneficial effect as adjuvant treatment after radical resection of primary ACC [17]. Experimental study suggested that mitotane may a sensitizer for radiotherapy [18] and most included articles for this meta-analysis have comprised patients using it. Gharzai et al. performed a retrospective propensity-matched analysis on 39 patients of ACC treated with ART. Results showed that LRFS and OS was significantly better with ART, compared with surgery only.

The results for our study cohort showed there were no significant difference between ART group and control group. However, we are the first systematic review comparing ART after resection and resection only in ACC to demonstrate a significant improvement in OS with ART (Fig. 3). In addition, our study shows that ART is strongly favorable for LRFS and RFS, with ORs of 4.08 and 2.27 respectively (Fig. 4&6). With ART after primary surgery, patients are therefore approximately four times more likely to be local recurrence-free than those treated with surgery only. This finding has the same results with two previous systematic reviews with meta-analyses [12, 16]. In terms of recurrence-free survival, debate exists as to whether ART after surgery is advantageous as compared to surgery only. One systematic review supported this notion [12], while the other does not find a significant difference [16]. With the inclusion of recent studies and our data, we demonstrate that treatment with ART is associated with improved recurrence-free survival in ACC. Overall, our results confirm the advantage of ART after primary surgery in OS, LRFS and RFS.

The advantages result from the progress in RT technique and machine, such as accurate delivery of disease site, and precise and consistent coverage of high-risk areas. Considering 25–30% of ACC patient may have inter-aortocaval lymph node metastases [19], this advance is beneficial to irradiate a wide territory. Besides, the RT adverse effects were considered tolerable and self-limited in all included studies. The main acute toxicity was nausea, fatigue, diarrhea with a low rate of potential kidney damage [12].

**Fig. 7:**
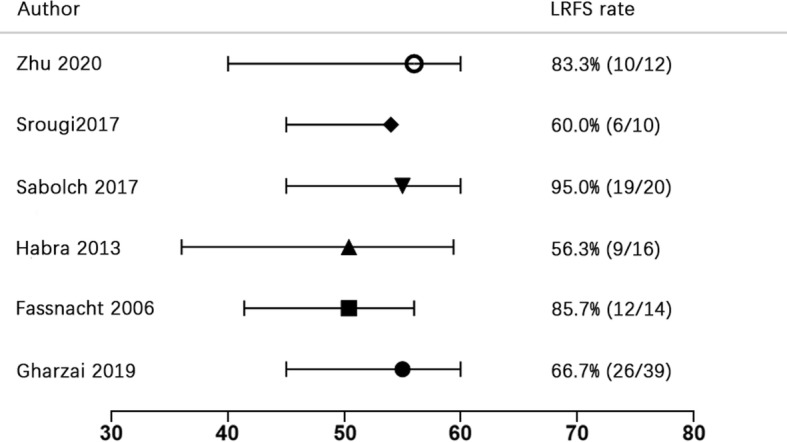
Radiation dose and LRFS rate of included studies

Regarding the ART dose, all the six retrospective studies used a median dose ≥50 Gy with a dose of 1.8–2.0 Gy per fraction (Fig. 7). The majority of patients was treated with conformal three-dimensional RT with only two patients reported to treated with conventional RT in the Germany study [9]. Two included studies did not describe the details on treatment technique, target volume, or treatment machine [10, 12]. It was difficult to observe a relation between the LRFS rate with RT dose or RT technique based on the limited available evidence.

The present study had a few limitations. Firstly, it is retrospective design and small sample size. Even matched some clinic characteristic in these retrospective studies, there are still unbalanced characteristics between the two groups of ART and non-ART patients, such as clinical condition and economic condition. On the other hand, due to the lack of standard guideline on the treatment of ACC, selection of the treatment discipline in different regions depends on the medical care or health insurance in different regions and experiences of the different medical centers. Therefore, randomized controlled trials are needed to evaluate the effect of ART in the treatment of ACC and minimize the selection bias. Secondly, different kinds of treatment plans and RT techniques are included in these studies, which may lead to heterogeneity in the meta-analysis. Although the ratios of mitotane usage are similar between groups in this meta-analysis, the initial treatment time and management of mitotane are different in all included studies, especially the opportunity of what is the optimal time to combined with RT. Last but not least, the lack of details about RT method also increased the heterogeneity of this study. Given the rarity of ACC and the limited literature about ACC with ART, our work provides relatively high-quality evidence.

## Conclusions

Patients who had under gone ART after primary resection have increased OS, LRFS and RFS compared to resection only. Our results suggest that ART may be a beneficial treatment modality for ACC because of the improved survival outcome. However, a larger-scale RCT would be urgently needed to prove these advantages.

## Data Availability

Owing to data privacy policy at our facility, publication of patient-related raw data is not possible.

## Abbreviations

ACC: Adrenocortical carcinoma
OR: Odds ratio
RT: Radiotherapy
ART: Adjuvant radiation
OS: Overall survival
RFS: Recurrence-free survival
LRFS: Locoregional recurrence-free survival
